# High-Intensity Gait Training With Functional Electrical Stimulation Enhances Corticospinal Excitability of Paretic Ankle Muscles in Individuals Post-Stroke

**DOI:** 10.1155/np/5529427

**Published:** 2025-12-01

**Authors:** Vyoma Parikh, Anjali Sivaramakrishnan, Justin Liu, Jiang Xu, Catherine F. Mason, Trisha M. Kesar

**Affiliations:** ^1^Division of Physical Therapy, Department of Rehabilitation Medicine, Emory University, Atlanta, Georgia, USA; ^2^Department of Physical Therapy, School of Health Professions, The University of Texas at San Antonio, Health Science Center, San Antonio, Texas, USA; ^3^Department of Medicine, Emory University, Atlanta, Georgia, USA; ^4^University of Science and Technology, Wuhan, China; ^5^Graduate Program in Neuroscience, Emory University, Atlanta, Georgia, USA

**Keywords:** electrical stimulation, gait, lower extremity, transcranial magnetic stimulation

## Abstract

**Introduction:**

High-intensity readmill training (FAST) and functional electrical stimulation (FES) are both evidence-supported interventions that improve gait function post-stroke, but their neural mechanisms are unclear. Here, we tested the hypothesis that FAST–FES training, which incorporates task-specific sensorimotor stimulation to paretic ankle muscles, would induce greater upregulation of lesioned corticospinal tract (CST) excitability compared to dose-matched training without FES in individuals post-stroke.

**Methods:**

In this repeated-measures crossover study, 11 participants >6 months post-stroke (66.25 ± 8.15 years, six females) received FAST–FES or FAST gait training protocols (comprising three training sessions) in a randomized order, with an intervening >3-week washout period. FES was applied to the paretic dorsi- and plantar-flexor muscles during the paretic swing and terminal stance phases of gait, respectively. CST excitability was measured before and after each training protocol from bilateral tibialis anterior and soleus muscles in three different test positions: sit–rest, sit–active, and quiet standing.

**Results:**

We found a significant main effect of intervention on training-induced change in motor evoked potential (MEP) amplitude (*p*=0.02). Post hoc comparisons revealed that FAST–FES caused a larger training-induced increase in MEPs than FAST training (*p*=0.01). FAST–FES did not affect CST excitability of the nonlesioned hemisphere, with no significant changes in MEP amplitude of the nonparetic ankle muscles.

**Conclusions:**

FAST–FES training increased corticospinal excitability in paretic ankle muscles without upregulating nonparetic ankle corticospinal drive, suggesting preferential induction of neuroplasticity in the lesioned CST.

## 1. Introduction

Stroke continues to be a leading cause of disability in the United States, with increasing incidence of stroke noted globally [1]. A majority of individuals post-stroke experience gait dysfunctions such as reduced walking speed and endurance, adversely affecting their quality of life and community participation [2–7]. Decreased paretic propulsion during late stance is an important biomechanical deficit contributing to slow walking speed and hemiparetic severity post-stroke [8]. Other impairments in ankle biomechanics include reduced dorsiflexion at heel strike or during swing phase, which are associated with foot drop and fall risk [9]. These ankle impairments are caused by reduced force generation by the paretic ankle dorsi- and plantar-flexor muscles [10, 11]. Thus, improving ankle muscle recruitment and normalizing ankle biomechanics during gait are important rehabilitation goals that can help enhance post-stroke walking function.

Treadmill training at a fast speed, FAST gait training, is an evidence-supported gait rehabilitation intervention commonly utilized to promote post-stroke gait recovery. FAST gait training provides high-intensity, high-repetition bilateral stepping practice [12–14]. Potentially, FAST training may induce bilateral neuroplasticity to both the lesioned and nonlesioned sensorimotor pathways, as stepping practice is not specifically targeted to the deficits of the paretic leg or the lesioned corticospinal pathways [15, 16]. Notably, while FAST treadmill-based gait interventions are increasingly used to improve clinical outcomes, training-induced modulation of neuromotor circuits with FAST gait training remain poorly studied, and need more investigation [17, 18].

Functional electrical stimulation (FES) is another popular rehabilitation intervention, applied either as a stand-alone treatment or in conjunction with other motor training programs. Because FES comprises electrical stimulation applied during a functional activity such as gait, FES can help retrain appropriate muscle activation patterns, promote motor learning, and enhance motor function [19–21]. FES may also promote neuroplasticity because the stimulation is delivered in a state-dependent and task-specific manner [22, 23]. Commercial FES systems for foot drop deliver motor-level stimulation to the ankle dorsiflexor muscles during the swing phase of the paretic limb, thereby augmenting voluntary activation through FES-assisted force [20, 23, 24]. FES also activates sensory nerve fibers, which enhance afferent ascending input to sensorimotor circuits, thereby increasing corticospinal tract (CST) output [25]. Task-specific timing of FES to multiple muscles (e.g., ankle dorsi and plantar flexors) at functionally appropriate points during the gait cycle may induce adaptive plasticity in spinal, spinocerebellar, and cortical circuits [26].

FAST–FES is a post-stroke gait intervention that pairs FAST treadmill training with phase-specific FES of the ankle dorsi- and plantar-flexor muscles. Previous studies support the biomechanical rationale for FAST–FES [14, 27–29]. FAST treadmill walking provides a biomechanical advantage by positioning the paretic trailing limb more posteriorly relative to the body's center of mass during the stance-to-swing transition, while also providing therapeutic benefits of high-intensity stepping practice. In conjunction with FAST, FES can increase force generation from the ankle plantar flexors during late stance phase to improve paretic propulsion, and from the ankle dorsiflexors during paretic swing phase to reduce foot drop [14, 28, 29]. Twelve weeks of FAST–FES gait training have been shown to enhance gait endurance and energy efficiency in people with chronic stroke, with sustained improvements noted 3 months post-training [3, 30, 31]. In recent studies, FAST–FES resulted in improvements in muscle activation of targeted ankle muscles, accompanied by improvements in gait biomechanics and walking function in individuals' post-stroke [14, 27, 29, 30]. Despite these benefits, similar to other gait rehabilitation interventions, FAST–FES suffers from high interindividual variability in the magnitude of training-induced gait improvements.

In addition to evaluating the effects of interventions such as FAST–FES on biomechanical and clinical outcomes, investigating if and how gait training interventions such as FAST–FES modulate sensorimotor neural circuitry is equally important. The primary motor cortex and the CST are important for motor function and recovery in individuals with stroke [32–35]. CST output can be assessed using motor evoked potentials (MEPs) elicited in response to transcranial magnetic stimulation (TMS) [36, 37]. However, a majority of research on neural correlates of rehabilitation focuses on upper extremity motor training paradigms, leaving a knowledge gap pertaining to neuroplasticity induced by clinically feasible gait retraining programs [38, 39]. Individuals post-stroke demonstrate reduced excitability of the lesioned CST. Limited previous research suggests that components of FAST–FES, such as high-intensity repetitive stepping practice [40, 41], and FES applied to the ankle dorsiflexor muscles for footdrop correction during gait, can modulate CST excitability [42, 43]. A recent study demonstrated that a single session of FES to ankle dorsi- and plantar-flexor muscles combined with gait training at self-selected speed improves CST excitability of paretic ankle plantar-flexor muscles, when compared to a gait training session without FES [43]. However, how FAST–FES modulates corticomotor circuits remains unclear, and merits systematic investigation [42, 44].

In this study, we aimed to evaluate the hypothesis that the short-term training-induced neural plasticity processes underlying FAST–FES may be more targeted to the lesioned corticospinal neural circuitry compared to FAST gait training. We tested 3 predictions related to the neural correlates of FAST–FES gait training. First, we predicted that 3 sessions of FAST–FES gait training would result in larger training-induced changes in lesioned CST excitability in paretic ankle muscles compared to FAST training. Second, we predicted that FAST–FES would induce larger training-induced changes in CST excitability of the paretic leg muscles compared to the nonparetic leg. Third, we predicted that training-induced changes in CST excitability would be correlated with improvements in gait speed.

## 2. Methods

The study protocol was approved by the Emory University Institutional Review Board, and all participants provided written informed consent. Participants were screened to confirm study eligibility, and written informed consent was obtained from all participants. Medical clearance was obtained, and a cardiac exercise stress test was performed to ensure participants could safely participate in gait training. Twelve participants with chronic stroke (age 66.25 ± 8.15 years; 6 females) were recruited for the study (Table 1).

Participant inclusion criteria were >6 months since onset of stroke, cortical or subcortical ischemic stroke, able to walk 10 m with or without an assistive device, sufficient cardiovascular health and ankle stability to walk on a treadmill for 2 min at a self-selected speed without orthosis, and resting heart rate 40–100 bpm. Exclusion criteria were hemorrhagic stroke, cerebellar signs (ataxic “drunken” gait or decreased coordination during rapid alternating hand or foot movements), inability to communicate with investigators, musculoskeletal, other medical conditions, or pain that limit walking, neglect/hemianopia, unexplained dizziness in last 6 months, neurologic conditions other than stroke, lack of sensation in lower limb affected by stroke, any medical diagnosis that would hinder the participant from walking or completing the experimental trial, contra-indications to TMS [45] such as metal implants in the head or face, history of seizures uncontrolled by medications, recurring or severe headaches/migraine, unexplained dizziness, syncope or nausea in the past 6 months.

Additionally, a physical therapist assessed walking function and lower limb impairments using the Fugl–Meyer Lower Extremity (FMA-LE) Scale, Berg Balance Scale, The Timed Up and Go Test, and overground gait speed (Table 1).

### 2.1. Study Design

**Figure 1:**
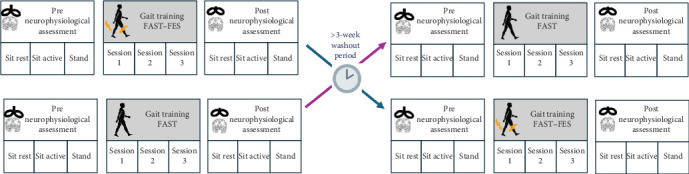
Overview of study protocol. Each participant completed two types of gait training protocols (FAST, FAST–FES) in a randomized crossover design, with a >3-week period in between. Each gait training protocol comprised three gait training sessions. For each type of gait training, neurophysiology assessments comprising measurement of CST excitability of paretic and nonparetic ankle muscles (in three different testing positions) using transcranial magnetic stimulation (TMS) were conducted in separate study visits before (pre) and after (post) the three gait training sessions.

This study utilized a repeated-measures crossover design. Participants were randomly assigned to receive 3 gait training sessions with either the FAST or the FAST–FES training protocol first, followed by the second set of gait training protocol sessions, with at least a 3-week washout period between the two sets of gait training (Figure 1).

### 2.2. Methods for Neurophysiologic Assessment of Corticospinal Excitability of Ankle Muscles

Before the start of gait training, participants underwent a study visit comprising pretraining neurophysiological assessment of CST excitability of the paretic and nonparetic ankle muscles. The same neurophysiologic assessment procedures were also performed at the post-training study visit after completing 3 training sessions, conducted on a separate visit within 1–3 days of the 3rd gait training session. Surface electromyography (EMG) sensors (2-cm diameter, Kendall Inc.) were attached to skin overlying the paretic and nonparetic leg soleus and tibialis anterior muscles. Accuracy of EMG electrode placement was confirmed, and maximal voluntary contractions were obtained from the target muscles for the tibialis anterior in a seated position, and for soleus in a standing position. Additionally, EMG activity in both tibialis anterior and soleus was recorded during 30 s of quiet standing. Measurements of CST excitability were performed for each participant under three different test conditions: sit–rest, sit–active, and quiet standing.

For measurement of paretic leg MEP responses in the sit–rest condition, participants were seated in a comfortable resting position on a chair with the hip and knee at approximately 90° angles, and feet supported firmly on the floor. The TMS hot spot for the paretic leg soleus muscle was determined as the site on the head that produced the maximal MEP amplitude at the lowest TMS intensity. Next, the resting motor threshold (RMT) was determined as the lowest TMS intensity at which an MEP amplitude >50 uV was elicited (Kesar et al. [14]). For the sit–active condition, participants were seated in a similar comfortable seated position and asked to maintain low-level voluntary soleus EMG activity equivalent to quiet standing, with visual feedback of ongoing EMG activity provided on a computer screen. For the sit–active condition, the active motor threshold (AMT) was defined as the lowest TMS intensity that elicited MEP amplitudes >100 uV. For quiet standing test conditions, participants were asked to stand in a quiet standing position with their feet about shoulder width apart. The same hotspot and AMT as determined in the sit–active condition was used during quiet standing. For all three test conditions, to collect MEP data, 10–20 TMS pulses were delivered at the soleus hotspot at a TMS intensity of 130% above the RMT (for the sit–rest test condition) or AMT (for the sit–active and quiet standing test conditions) [14].

A similar protocol was used to record MEPs from the nonparetic tibialis anterior and soleus muscles in a sit–rest condition, with TMS delivered to the nonparetic soleus hotspot at an amplitude of 130% above the RMT for the nonparetic soleus.

### 2.3. Methods for Gait Training

The FAST and FAST–FES gait training protocol procedures were consistent with previous publications [3, 14, 30, 46, 47]. Gait training was performed as participants walked on a split-belt treadmill instrumented with two force platforms (AMTI Inc., Watertown, MA). Each gait training period comprised of 3 gait training sessions over a 1–1.5-week period (2–3 sessions per week). Each gait training session included total 30 min of treadmill walking, comprised of five 6-min treadmill walking bouts with rest breaks between bouts. The last training bout (bout 6) comprised 6-min of over ground walking during which subjects were asked to walk as FAST as they could safely and practice the gait patterns learned on the treadmill. A physical therapist implemented all gait training sessions, with help from 1 to 2 research assistants.

Gait speed for FAST and FAST–FES training was determined as a speed faster than the participant's self-selected speed on the treadmill, and a FAST speed that the participants perceived they could maintain for ~4-min of continuous walking. The training speed was progressed within and across sessions as appropriate, in a personalized manner to increase training intensity for each participant based on heart rate, exertion, participant preference, and the physical therapist's judgement. For the same participant, the average training speeds were matched for both types of gait training (FAST and FAST–FES), such that similar training speeds were used in both the gait training conditions.

To ensure safety during gait training, participants wore a harness suspended from the ceiling without any body weight support, and participants were allowed to hold a front handrail during walking. Participants' heart rate was monitored during training using a heart rate sensor placed on the chest under clothing (Polar USA, Lake Success, NY), and their fatigue level was monitored using the rating of perceived exertion scale. The structure and dosage of the FAST and FAST–FES training sessions were similar, with the only difference being the introduction of FES during the FAST–FES sessions. During FAST training, no verbal instructions were provided to the participants unless needed for maintaining safety during gait training. During FAST–FES training, surface electrical stimulation pads were attached to the paretic tibialis anterior muscle (50.8 mm × 50.8 mm electrodes, TENS Products, CO) and gastroc–soleus muscle bellies (76 mm × 127 mm electrodes, ConMed, NY). A Grass S8800 stimulator in combination with an SIU8 stimulus isolation unit was used to deliver electrical stimulation (Grass Instruments, MA). Two footswitches (forefoot and hindfoot; 25-mm MA-153, Motion Lab Systems Inc., LA) were attached to the soles of both shoes to enable closed-loop control of the timing of FES delivery (CompactRIO, National Instruments, TX) [20, 48, 49]. Stimulation was delivered to the ankle dorsiflexors when the participant's foot was in swing phase, and to the ankle plantar flexors during the late stance phase of gait (between paretic heel-off and toe-off). Stimulation parameters were 60 μs pulse duration, 30-Hz variable frequency, and train duration based on gait cycle timing. FES amplitude was determined at the start of every training session as motor-level stimulation that elicited appropriate functional movements at the ankle, or maximal tolerance, whichever occurred first [47, 49]. For the ankle dorsiflexors, the desirable FES intensity goal was ankle dorsiflexion to neutral when seated with the leg suspended. For the gastroc–soleus, the desirable FES intensity goal was to lift the paretic heel off the ground during standing in a staggered stance similar to paretic leg terminal double support [20]. FES was delivered intermittently during gait training, with alternating 1-min periods with and without FES for every 6-min gait training bout to prevent fatigue and dependence on stimulation and encourage volitional activation with FES and promote motor learning [50]. Before training, participants were given verbal instructions to explain the functional role of the dorsi- and plantar-flexor FES and were instructed to use their own volitional force to “help the stimulation using their own muscle force” when the FES was on, and to continue to practice volitionally the movement that the FES was training during intervening training periods without FES.

### 2.4. Data Processing and Statistical Analyses

Surface EMG data were collected using a BIOPAC system, which allowed for the accurate recording of muscle electrical activity. The peak-to-peak MEP amplitude was measured for each muscle and averaged over 20 individual MEPs to ensure a reliable and representative value for analysis.

Descriptive statistics, including means and standard deviations, were calculated for the demographic variables. Normality and outliers were assessed using Kolmogorov–Smirnov tests and Q–Q plots to confirm that parametric analysis assumptions were met. All statistical analyses were performed using SPSS (Version 29.0).

To evaluate our first hypothesis related to training-induced change in CST excitability on the paretic leg, a 3-way repeated measures ANOVA was used to assess the effects of gait training protocol (2: FAST, FAST–FES), task condition (3: sit–rest, sit–active, standing), and paretic leg muscles (2: TA, soleus) on training-induced change in MEP amplitude (post minus pre). To assess our second hypothesis regarding whether the effects of the gait training condition differ between the paretic and nonparetic side, a 3-way repeated measures ANOVA was conducted to assess the effect of training intervention (2: FAST, FAST–FES), leg (2: paretic, nonparetic), and muscle (2: TA, soleus). Appropriate post hoc analyses with corrections for multiple comparisons were performed if a significant interaction was observed. Alpha levels were set at 0.05. In the absence of significant interactions, data were pooled across muscles and test conditions to assess intervention effects.

In addition, a priori planned paired *t*-tests were performed to evaluate the effect of each gait training protocol (FAST and FAST–FES training sessions) on training-induced change in MEP amplitude in the paretic leg. We also performed a priori planned paired *t*-tests to compare training-induced change in MEP amplitude for FAST and FAST–FES training conditions on paretic vs. nonparetic muscles, to test our hypothesis focused on comparing the stimulated (paretic) vs. nonstimulated (nonparetic) muscles. To test our third hypothesis, we conducted Pearson's correlation analyses to evaluate associations between training-induced change in MEP amplitude vs. change in gait speed. To evaluate the effects of training on overground gait speed, paired *t*-tests were used to compare overground fast gait speeds at pre vs. post-timepoints for both FAST and FAST–FES training conditions, and to compare training-induced change in gait speed between FAST vs. FAST–FES. To supplement the above analyses, we also reported the magnitude of standardized effect sizes [51], as well as evaluated the proportion of participants who showed larger improvements with FAST–FES vs. FAST.

## 3. Results

Data were obtained on a total of 12 participants with chronic stroke (age = 66.25 ± 8.15 years; time post-stroke = 95.25 ± 45.23 months) (Table 1). All participants completed both gait training conditions, comprising three sessions of FAST and FAST–FES gait training, as well as pre and postneurophysiology evaluations for each type of gait training condition. One participant was an outlier (>2 standard deviations of the average change in MEP amplitude), likely due to measurement error, and their data were excluded from the analyses to maintain consistency in the study population.

### 3.1. Comparison of the Effects of FAST and FAST–FES Gait Training on Corticospinal Excitability of Paretic Ankle Muscles

The repeated-measures ANOVA evaluating the effect of intervention (FAST, FAST–FES), task condition (sit–rest, sit–active, standing), and paretic muscle (paretic soleus, paretic TA) on training-induced change in MEP amplitudes showed a significant main effect of intervention (*p*=0.02^*∗*^, *F* [1, 8] = 8.58, effect size *np*^2^ = 0.52), no significant main effect of task condition (*p*=0.40, *F* [2, 16] = 0.96, *np*^2^ = 0.11), no main effect of muscle (*p*=0.44, *F* [1, 8] = 0.68, *np*^2^ = 0.08), and no significant interaction effects (*p*=0.72, *F* [2, 16] = 0.34, *np*^2^ = 0.04) (Figure 1).

**Figure 2:**
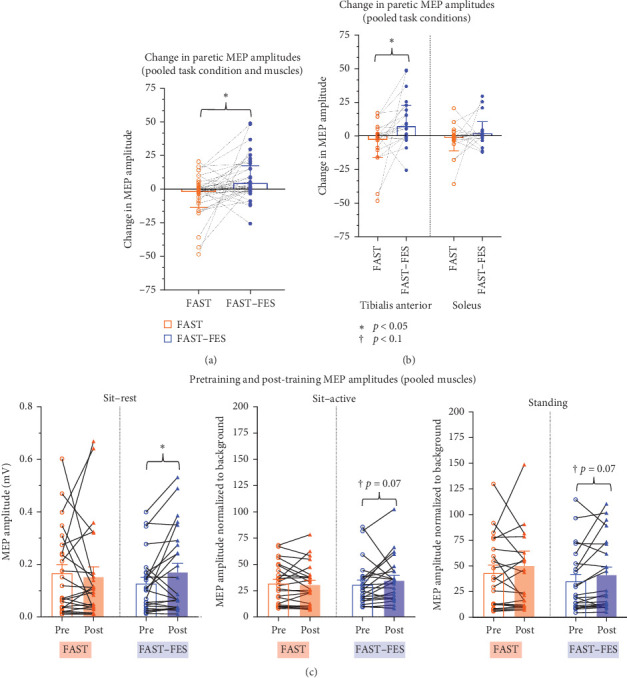
Effects of FAST and FAST–FES gait training protocols on MEP amplitudes of paretic ankle muscles. Training-induced change in MEP amplitudes (post − pre) of the paretic limb were compared for the FAST and FAST–FES training when both the task/testing condition and muscles were pooled (A), as well as separately for tibialis anterior and soleus muscles when the task conditions were pooled (B). We also show the MEP amplitudes at pre- and post-time points for each task condition, pooled across both muscles (C). Note that FAST–FES training induced a significantly larger change in MEP amplitude compared to the FAST training (pooled for both muscles and task conditions (A), and for the tibialis anterior muscle (B). Also, FAST–FES training, but not FAST training, resulted in a significant increase in MEP amplitude from pre to post in the sit–rest condition and showed a statistical trend for a significant increase in the sit–active and standing conditions (C).

Because there were no significant interaction effects in the ANOVA and no significant main effects of task condition or muscle, the data for the three task conditions and the two muscles were pooled for subsequent post hoc comparisons. The paired post hoc *t*-test comparing training-induced changes in MEP amplitude of the paretic leg (data pooled for both muscles and across three task conditions) showed that FAST–FES training resulted in a significantly greater increase in paretic leg MEP amplitudes compared to the FAST training (*p*=0.01^*∗*^, Cohen's *d* = 0.60). To evaluate differential effects of gait training on the paretic TA and soleus muscles individually (data pooled across the three task conditions), paired *t*-tests showed that compared to FAST training, FAST–FES training induced significantly larger increases in TA MEP amplitudes (*p*=0.01^*∗*^, Cohen's *d* = 0.69), but there were no significant differences between FAST–FES and FAST for soleus MEP amplitudes (*p*=0.26, Cohen's *d* = 0.48) (Figure 2).

### 3.2. Comparison of the Effects of FAST–FES and FAST Gait Training on Corticospinal Excitability of Paretic vs. Nonparetic Ankle Muscles

**Figure 3:**
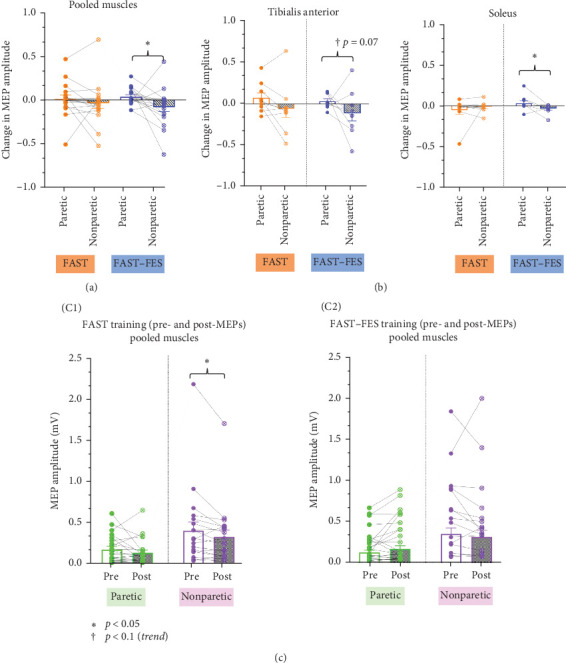
Effects of FAST and FAST–FES training on paretic and nonparetic MEP amplitudes. The training-induced change in MEP amplitude (post − pre) was compared between the paretic and nonparetic limb (sit–rest condition) for the FAST and FAST–FES training conditions when the tibialis anterior and soleus muscles were pooled (A), as well as separately for tibialis anterior and soleus muscles (B). We also compared pre- vs. post-MEP amplitudes for both muscles, pooled for paretic and nonparetic limb (C), for FAST training (C1) and FAST–FES gait training (C2). Our results showed that FAST–FES, but not FAST training, resulted in a significant difference between paretic vs. nonparetic limb when muscles were pooled (B), and for the soleus muscle when the muscles were not pooled (C). Also, FAST training resulted in a significant reduction in MEP amplitude on the nonparetic limb.

The repeated measures ANOVA evaluating the effects of intervention (FAST, FAST–FES), stimulated side (paretic, nonparetic), and muscles (TA, soleus) on training-induced change in MEP amplitudes showed a significant main effect of stimulation side (paretic vs. nonparetic) (*p*=0.01^*∗*^, *F* [1, 9] = 9.74, *np*^2^ = 0.52, power = 0.79), and a significant interaction between muscle and stimulated side (*p*=0.03^*∗*^, *F* [1, 9] = 6.77, *np*^2^ = 0.43). There was no main effect of intervention (*p*=0.85, *F* (1,9) = 0.04, *np*^2^ = 0.01), no main effect of muscle (*p*=0.73, *F* [1, 9] = 0.13, *np*^2^ = 0.01), and no significant 3 way-interaction between intervention, stimulation side, and muscle (*p*=0.94, *F* [1, 9] = 0.01, *np*^2^ = 0.001) on training-induced change in MEP amplitudes (Figure 3).

Planned paired *t*-tests showed that FAST–FES training resulted in a significant increase in MEP amplitude of the paretic leg ankle muscles (pooled TA and soleus) compared to the nonparetic leg ankle muscles (*p*=0.04^*∗*^, Cohen's *d* = 0.45). In contrast, following FAST training, there were no significant differences in training-induced changes in MEP amplitudes between the paretic and nonparetic leg muscles (paretic leg DMEP = −32 mV, nonparetic leg DMEP = −63 mV, *p*=0.29, Cohen's *d* = 0.14).

Planned paired *t*-tests showed that following FAST training, there was a significant decrease in the MEP amplitude for the nonparetic limb between pre and post training (TA and soleus pooled) (*p*=0.02, Cohen's *d* = 0.50) but not for the paretic limb (*p*=0.20). FAST–FES did not result in significant decrease in MEP amplitude on the nonparetic limb (*p*=0.39, Cohen's *d* = 0.28). FAST–FES resulted in an increase in MEP amplitude on the paretic limb with a medium effect size, but change was not statistically significant (*p*=0.22, Cohen's *d* = 0.45).

### 3.3. Additional Results

**Figure 4:**
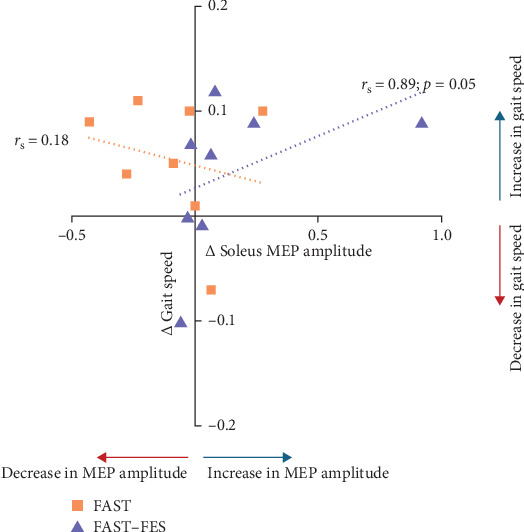
Correlation between gait training-induced change in MEP amplitude and change in walking function. For FAST–FES training, the training-induced change in soleus MEP amplitude showed a significant positive correlation with training-induced change in overground fast gait speed. Note that the majority of participants showed a positive change in MEP amplitude and gait speed with FAST–FES gait training, whereas for FAST training, there was a negative change in MEP amplitude (i.e., decreased corticospinal excitability) for the majority of participants, despite a training-induced increase in gait speed.

Paired *t*-tests showed a significant increase in overground fast gait speed from pre to post training for FAST–FES (*p*=0.02, Cohen's *d* = 0.21), but not FAST (*p*=0.07). However, there were no significant differences in the training-induced change in overground fast gait speed between FAST–FES vs. FAST gait training (*p*=0.3). Correlation analysis showed a significant positive correlation between training induced change in soleus MEP amplitude (during quiet standing) and change in overground gait speed for FAST–FES training (*r*_*s*_ = 0.89, *p*=0.005), but no significant correlation was noted for FAST training (*r*_*s*_ = 0.18, *p*=0.67) (Figure 4).

**Figure 5:**
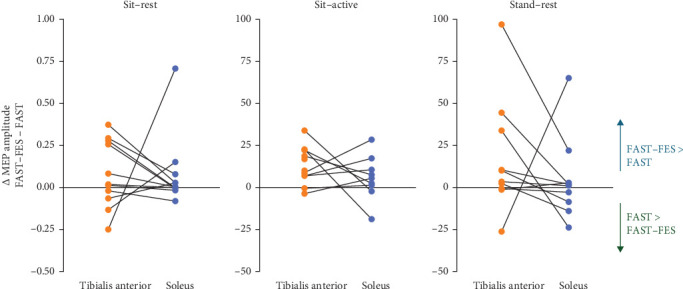
Difference in training-induced changes in MEP amplitude for FAST–FES vs. FAST. The majority of participants exhibited a positive and/or greater magnitude of change in MEPs after FAST–FES compared to FAST for both the tibialis anterior and soleus muscles across all test positions.

As an additional descriptive analyses, we evaluated the difference between the training-induced MEP amplitude change (Post minus Pre training) between FAST–FES and FAST by calculating the difference between the FAST–FES vs. FAST MEP change score, such that a positive value would indicate an advantage for FAST–FES. A majority of participants showed a positive change in MEP amplitude with FAST–FES compared to FAST training for the tibialis anterior (six out of 10 for sit–rest, eight of 10 for sit–active, and eight of 10 for quiet standing) and soleus muscles (seven out of 10 for sit–rest, eight of 10 for sit–active, and six of 10 for quiet standing), demonstrating superiority of FAST–FES training with respect to effects on CST excitability (Figure 5).

## 4. Discussion

The current study compared the effects of two gait training protocols, FAST–FES and FAST, on corticospinal excitability of the paretic and nonparetic leg soleus and tibialis anterior muscles in individuals post-stroke. Our results suggest that FAST–FES gait training may enhance the excitability of corticospinal connections to the paretic limb to a larger extent and with greater specificity than FAST training without FES. In this study, we tested 3 predictions related to the neural correlates of FAST–FES gait training. Our first prediction was that 3 sessions of FAST–FES gait training would result in larger training-induced changes in CST excitability in paretic ankle muscles compared to FAST. Our results were consistent with this prediction, showing that compared to FAST training, the FAST–FES training resulted in a significant increase in paretic leg MEP amplitude when all the test conditions and muscles were pooled. Notably, for MEP amplitudes pooled across all 3 test conditions (sit–rest, sit–active, standing), FAST–FES resulted in a larger training induced increase in MEP amplitude in the tibialis anterior muscle compared to FAST training, but not for the soleus muscle. Our second prediction was that FAST–FES will result in larger training-induced changes of CST excitability in the paretic vs. the nonparetic leg. Consistent with this prediction, our results showed that FAST–FES resulted in a significantly larger training-induced change in MEP amplitudes for the paretic vs. nonparetic ankle muscles. In contrast, training-induced change in MEP amplitude between the paretic and nonparetic limb muscles did not differ for FAST training. Interestingly, FAST training resulted in a significant reduction in the MEP amplitude in the nonparetic lower-limb muscles between pre and post-training, when the data for both muscles were pooled, whereas FAST–FES training did not significantly change the MEP amplitude in the nonparetic limb. Our third prediction was that training-induced changes in CST excitability would be correlated with improvements in gait speed, and our correlation results were consistent with this hypothesis for FAST–FES training.

Our results showed that FAST–FES training increases CST excitability of the lesioned hemisphere, as indicated by significant increases in MEP amplitude of the paretic ankle muscles. In contrast, FAST training, which was a dose-matched control training without FES, did not show such an increase in paretic CST excitability. Our findings are similar to a previous study by Palmer et al. [43], which showed that a single session of FES to ankle dorsi- and plantar-flexor muscles combined with treadmill training at self-selected speed increased CST excitability of the paretic soleus muscle. Similar to our results involving FES to two muscles (ankle dorsi and plantar flexors), previously, FES applied to the target only ankle dorsiflexor muscles has been shown to increase CST excitability of the target muscles in able-bodied individuals [42, 44]. FES-induced enhancement of corticospinal output may be attributed to the targeted, task-specific activation provided by sensorimotor stimulation to the paretic ankle dorsi and plantar flexors [27], which results in the upregulation of the excitability of the corresponding cortical somatosensory and M1 representations of the stimulated muscles [43]. Notably, during FAST–FES, FES was delivered intermittently and not continuously, and participants were instructed to use their own volitional force to “help the stimulation” during periods with and without FES. Thus, the FAST–FES training protocol was designed to promote engagement and volitional activation of the participants' own corticospinal pathway during stepping practice, using FES as a sensorimotor cue or “tutor” regarding when and how much to activate the dorsi- and plantar-flexor muscles. Furthermore, the pairing of the descending corticospinal volleys generated by volitional contractions with ascending sensory activation generated by the FES could result in strengthening of cortico–cortical and cortico–motoneuronal circuits, contributing to larger TMS-induced MEPs following FAST–FES training.

In this study, the FAST gait training condition was a “negative control” dose-matched with the active FAST–FES condition, by incorporating FAST walking in the absence of stimulation. FAST training involves bilateral repetitive stepping practice, which has been shown to induce improvements in walking function [40, 41, 52]. During clinical practice, FAST training may be augmented by verbal instructions or feedback provided by the clinician during stepping practice, which may enhance the specificity with which FAST training targets activation of muscles in the paretic leg [53]. However, in the current study, during FAST training, we did not provide instructions to participants to specifically target the paretic leg, which could be why FAST training did not induce a change in lesioned M1 excitability. Potentially, augmenting FAST training with verbal instructions or visual biofeedback to encourage volitional activation may be a valuable comparison to FAST–FES, and merits future investigation.

During FAST–FES, despite FES being delivered to both the tibialis anterior (during swing phase) and soleus muscles (during late stance phase), the tibialis anterior MEPs demonstrated significant training induced increases with FAST–FES training, but the soleus MEPs did not. Compared to FAST, FAST–FES resulted in greater training-induced change in MEPs of the tibialis anterior muscle, but not for the soleus muscle. Notably, for measurement of CST excitability, TMS was delivered to the hotspot of the soleus muscle at an intensity based on soleus motor threshold, as this approach enables the collection of MEPs from both the tibialis anterior and the soleus muscles simultaneously [54]. Interestingly, similar to our results, a previous study by Kaneko et al. [55] reported that a single session of FES applied to the dominant tibialis anterior and soleus muscle, combined with active observation and motor imagery of walking in able-bodied adults, resulted in increased MEP amplitude in the tibialis anterior muscles, but not in the soleus muscle. In the previous study, FES was applied to the tibialis anterior and soleus muscle in a seated position and timed to coincide with prerecorded EMG activation of the dorsi and plantar flexors during gait. One plausible explanation could be that compared to soleus, the tibialis anterior has greater strength and number of corticomotoneuronal connections, and perhaps greater capacity for CST plasticity [56, 57]. Also, during gait, the soleus muscle activity during the terminal stance phase may be mediated by spinal, subcortical, and cortical neural circuits to generate rapid and high forces for propulsion, with less specific task requirements for individuation and force accuracy. On the contrary, tibialis anterior activity is needed to clear the foot off the ground during the swing phase, which requires more specific and fractionated motor cortical control of ankle position and toe-clearance. Potentially, different roles of the tibialis anterior and soleus during gait could relate to differences in the magnitude of motor cortical control needed for the two muscles [57, 58], as well as their capacity for gait training-induced plasticity. Intermuscle differences in training-induced plasticity and dose–response are important considerations for future research, and can inform the design of FES strategies involving stimulation of multiple muscles, beyond the conventional and simpler approach of dorsiflexor FES for footdrop correction.

In our study, we conducted TMS assessments across sit–rest, sit–active, and quiet standing conditions, which helps provide methodological redundancy across our neurophysiologic outcomes and enhances rigor [59]. In the past, for lower extremity muscles, TMS assessments have been commonly performed in a seated position. However, recent evidence supports lower limb TMS assessments in the standing position, due to heightened engagement of neural pathways and the task-specificity of neuroplasticity [15, 54, 60]. Previously, in young able-bodied adults, TMS measurements of the soleus in a standing coactivation position yielded larger and more consistent MEP responses compared to those in the seated posture [60]. In individuals post-stroke, there is evidence of coactivation of the agonist and antagonist muscles in the paretic limb, and conducting TMS assessment in standing positions in future studies may enable more robust and task-relevant outcome measures.

Another important result from the current study was the demonstration that the FAST–FES gait training protocol may preferentially enhance the excitability of corticospinal connections to the paretic limb. FAST–FES gait training resulted in a significantly greater change in MEP amplitude of the paretic leg ankle muscles (pooled TA and soleus) compared to the nonparetic ankle muscles. On the other hand, there were no significant differences between change in MEP amplitude of the paretic and nonparetic ankle muscles with FAST gait training. The targeted effect of FAST–FES on the paretic leg may confer important mechanistic and therapeutic advantages that support its clinical use. In recent studies, FES applied to the paretic limb has been shown to improve target paretic muscle recruitment (measured using EMG) and increase CST excitability [27, 42, 43]. FAST training provides bilateral stepping practice to both paretic and nonparetic muscles [12–16]. Thus, combining FAST with FES likely results in an increased recruitment of the paretic tibialis anterior and soleus muscles during gait training, and thus help increase the lesioned CST excitability with greater specificity. FAST training did not change paretic limb MEP amplitude, but significantly reduced MEP amplitude of the nonparetic limb muscles. On the contrary, FAST–FES did not result in a significant change in the nonparetic limb MEP amplitude. Acute reduction in CST excitability with high-intensity gait training in stroke, specifically in the nonparetic limb muscles has been shown previously [17]. Madhavan et al. [17] reported that high-intensity treadmill training reduced the CST excitability in both the paretic and nonparetic tibialis anterior in individuals post-stroke. This downregulatory effect on the paretic limb was mitigated when gait training was paired with excitatory tDCS targeting the lesioned hemisphere motor cortex [17]. Other studies have also shown that combining a cortical priming intervention such as anodal tDCS to the lesioned hemisphere or an ankle tracking task with high-intensity treadmill training may upregulate cortical activity for the paretic tibialis anterior representation [61, 62]. One potential reason for the FAST-induced reduction in excitability in the nonparetic leg has been hypothesized to be the development of central fatigue with high-intensity training, which can reduce descending motor output [63].

Demonstrating the behavioral relevance of training-induced change in MEP amplitude, we showed that these training-induced increases in CST excitability were correlated to training-induced improvements in overground gait speed for FAST–FES gait training, but not FAST training. FAST–FES significantly improved CST excitability in the paretic limb, and these neurophysiologic changes were accompanied by improvements in gait function. Similarly, previous studies have shown gait training to result in associated neural adaptation as assessed by CST excitability in individuals post-stroke [18, 43, 64]. FAST–FES has been shown to have a beneficial effect gait speed [3, 4, 14, 27, 43, 65]. On the contrary, FAST training-induced changes in CST excitability were not correlated with gait outcomes. Note that the 3-session effects of FAST–FES on gait speed shown here were observed despite this being a relatively short exposure to the gait training intervention. Conventional gait rehabilitation research studies and clinical practice incorporate a greater number (e.g., 8–18) of training sessions. Potentially, the short-term changes in corticospinal neurophysiology during the first 3 sessions of gait training may provide valuable data to determine whether the participant is likely to benefit from continuing the same rehabilitation program for another 6–12 sessions. Neurophysiologic outcomes at baseline and short-term changes (e.g., after 3 training sessions, as shown here) may provide biomarkers predicting long-term treatment response, help identify potential responders, and thus inform clinical decision-making.

We would like to acknowledge a few limitations in our study. We tested a small sample of chronic stroke survivors (*n* = 12); however, the results from this study detected significant differences and can serve as important preliminary findings to inform future investigations of the neural mechanisms of post-stroke gait training. In small sample studies, statistical significance does not necessarily indicate a robust or reliable treatment effect [66], can reduce the likelihood that the observed effect reflects a true underlying effect, and may inflate effect size estimates [67, 68]. We employed a cross-over repeated-measures study design to help address the limitations of the small sample size. Here, CST excitability was assessed in three different test positions, which helps enhance rigor by evaluating the output of the CST in a task-specific manner, but may induce fatigue due to the longer duration of test sessions. However, sufficient rest breaks were provided to minimize the effect of fatigue on our results [46, 60, 69]. Here, we focused on evaluating the effects of 3 sessions of gait training, but did not assess long-term retention of training-induced changes in behavior or CST excitability. However, our study design was appropriate and rigorous for our mechanism-focused study goals of understanding neural mechanisms of FAST and FAST–FES.

## 5. Conclusions

Our results showed that compared to FAST gait training without FES, FAST–FES training may provide the advantage of inducing greater enhancement of corticospinal output, preferentially targeting and upregulating the lesioned (without modulating the nonlesioned) CST. We posit that due to these beneficial neurophysiologic effects of FAST–FES shown in our current study, even if the effects of FAST and FAST–FES on behavioral outcomes are equivalent, the addition of FES can still provide an advantage in clinical rehabilitation. Additionally, the increase in CST excitability with FAST–FES training was significantly correlated with improvement in gait function. Our study lays the foundation for elucidating the neural mechanisms of clinically relevant gait training interventions, with the long-term goal of determining why and for whom such interventions are most effective.

## Figures and Tables

**Table 1:** Participant demographics and clinical characteristics at baseline.

Participant number	Sex	Age (years)	Time since stroke (months)	Side of hemiparesis	Gait speed (m/s)	FAST: overground fast gait speed change (m/s)	FAST–FES: overground fast gait speed change (m/s)	Berg balance scale score (max score = 56)	Timed up and go test (s)	Fugl–Meyer score (max score = 34)
1	*F*	69	114	Right	0.69	0.12	0.10	48	13.41	28
2	*F*	71	113	Left	0.52	0.09	0.04	48	20.49	25
3	*F*	49	76	Left	0.73	0.06	0.01	47	14.27	31
4	*M*	69	217	Right	1.22	0.09	0.11	54	7.86	39
5	*M*	78	72	Left	0.81	0.00	0.09	54	11.8	26
6	*F*	64	62	Right	0.12	0.00	0.05	34	79.05	16
7	*F*	77	66	Right	0.59	0.07	−0.07	43	14.48	17
8	*M*	67	57	Left	0.93	−0.10	0.10	55	9.33	28
9	*M*	63	88	Left	0.46	–	–	42	21.98	23
10	*M*	57	64	Right	0.88	–	–	56	–	27
11	*M*	61	135	NA	–	–	–	51	–	14
12	*F*	70	79	NA	1.09	–	–	52	–	25
Average	–	66.25	99.25	–	0.73	0.04	0.05	48.67	21.41	24.92
SD	–	8.15	45.43	–	0.31	0.07	0.06	6.49	22.10	6.91

*Note:* –, missing data.

Abbreviations: *F*, female; *L*, left; *M*, male; *R*, right.

## Data Availability

The data that support the findings of this study are available from the corresponding author upon reasonable request.
